# Supplementary material for: Beyond coverage: Can vaccination reduce child mortality despite structural inequality? A global ecological analysis of routine childhood immunization (2010–2023)
Source: PLoS One. 2026 May 18;21(5):e0340756. doi: 10.1371/journal.pone.0340756 (PMC13183232; doi:10.1371/journal.pone.0340756)
Supplement: S1 File — (DOCX) [file pone.0340756.s001.docx]

**Manuscript PONE-D-25-68661**

Dear Editors and Reviewers:

Thank you for the opportunity to submit a revised version of the manuscript “Beyond coverage: can vaccination reduce child mortality despite structural inequality? A global ecological analysis of routine childhood immunization (2010–2023)” for consideration in PLOS One. We are grateful for the time and effort provided, and we sincerely appreciate the feedback that has helped us to strengthen our manuscript.

We have implemented the recommendations, and the revised manuscript highlights the corresponding changes.

**Reviewers**' **Comments to the Authors:**

**Reviewer 1**

1. Comment from Delete duplicate “for six” (line 78)

**Author response:** Thank you for this observation. The duplicated phrase has been removed from the revised manuscript (line 78)

2. Comment to change “accounting country level“ to “accounting for country-level” (line 76) for grammatical accuracy

**Author response:** Thank you for bringing this to our attention. We have revised the phrase to “accounting for country level” in line 76.

3. Consider brief vaccine descriptions, noting targeted diseases, efficacy evidence, and known disparities in uptake amid structural inequalities.

**Author response:** Thank you for this valuable suggestion. A brief description of the vaccines included in the analysis, including the diseases they target and evidence supporting their effectiveness in reducing child mortality, has been added to the Data sources and variables section of the revised manuscript.

“Vaccination coverage indicators included the proportion of children receiving Bacillus Calmette-Guérin (BCG), which protects against severe forms of childhood tuberculosis; the second dose of measles-containing vaccine (MCV2), targeting measles infection and typically administered during the second year of life (around 15 to 18 months); a later schedule is associated with an increased risk of missed follow-up visits and drop-out between measles vaccine doses in routine immunization programs [21-22]; and the third dose of diphtheria-tetanus-pertussis (DTP3), hepatitis B (HepB3), *Haemophilus influenzae* type b (Hib3), and poliovirus (Pol3) vaccines, which prevent major infectious diseases such as diphtheria, tetanus, pertussis, hepatitis B infection, invasive *Haemophilus influenzae* type b disease, and poliomyelitis, respectively. These antigens are the core components of routine child immunization programs and have contributed substantially to global reduction in vaccine-preventable child mortality. Despite this effectiveness, vaccination coverage remains uneven across countries, reflecting structural inequalities, including socioeconomic disadvantage, geographical barriers to healthcare access, and variations in health system capacity, particularly in low-income settings.”

4. Comment from Minor suggestions:

- Clarify missing data exclusion threshold
- Add statistical software and model diagnostics
- Elaborate COVID-19 disruption handling.

**Author response:** Thank you for these beneficial suggestions. We have addressed these points in the revised manuscript, specifically in the Materials and Methods section.

“To ensure data quality and comparability, the integrity of country-year observations was evaluated across all variables; the number of missing values (NAs) per country ranged from 84 to 540 across the study period. Countries with more than one-third of the maximum observed number of missing values (>180 NAs) were excluded from the analysis. This threshold was applied to minimize bias from excessive missing data while the final analytical sample was restricted to countries meeting this inclusion criterion.”

“ In addition, because routine immunization services were widely disrupted during the COVID-19 pandemic, the study period (2010-2023) was intentionally retained to capture both pre-pandemic trends and pandemic-related deviations in vaccine coverage. Rather than excluding these observations, country-year data from 2020 to 2023 were maintained to evaluate changes in coverage trajectories. Temporal trends were, therefore, interpreted considering this global disruption, which allowed the analysis to capture both the abrupt decline in vaccination coverage in 2020 and the subsequent partial recovery during 2021-2023.”

“All statistical analyses were conducted using R version 2024.04.2. Model assumptions were evaluated through standard diagnostic procedures, including inspection of residual distributions and assessment of multicollinearity among predictors.”

5. Line 324: Change “suggest” to “suggests.”

**Author response:** Thank you for pointing this out. The correction has been implemented in the revised version of the manuscript.

6. Elaborate briefly on MCV2’s later scheduling vulnerability.

**Author response:** Thank you for this suggestion. A brief explanation regarding the later scheduling of MCV2 and its potential vulnerability to missed vaccinations or service disruptions has been added to the Data sources and variables section of the revised manuscript.

“ … the second dose of measles-containing vaccine (MCV2) targets measles infection and is typically administered during the second year of life (around 15 to 18 months); a later schedule is associated with an increased risk of missed follow-up visits and drop-out between measles vaccine doses in routine immunization programs [21-22].”

7. Line 360: Change “highlights” to “highlight.”

**Author response:** Thank you for pointing this out. The correction has already been made in the revised version of the manuscript.

**Reviewer 2**

Dear Authors,

Thank you for your work. Please find below, some minor remarks you might like to address:

1. Line 77 "for six" repeats

**Author response:** Thank you for pointing this out. The correction has already been made in the revised version of the manuscript.

2. The sentence starting on line 224 "Differences across..." needs to be rewritten because currently it looks like the p-value refers to BCG vaccination coverage, while you state it is the exception.

**Author response:** We agree with the reviewer’s suggestion. The correction has already been made to make it clearer that we wanted to state it was the exception.

3. Line 253, all ⍴ < 0.001 - you should substitute the rho symbol with p as you are stating a p-value.

**Author response:** Thank you for pointing this out; the rho symbol has already been replaced with “p”.

4. Line 288 - S1 and S2 are cited in the text so they shouldn't appear in the supplementary material section. Once removed numbers should be adjusted to match numbers in the supplementary materials file.

**Author response:** The reviewer does not clarify if they are referring to S1/S2 Table or S1/S2 Figure; however, none of them are cited in the text, especially not in line 288, so we are not sure of what the reviewer is referring to.

5. The csv-file (Table S1. Country–year analytical dataset (2010–2023). is not accessible when downloading the supplementary materials.

**Author response:** The table the author is referring to is Table S3 in the CSV file. The code writes two CSV files that can be opened in Excel so that they are understandable. The code writes two CSV files that can be opened in Excel in order for it to be understandable.
